# Nanoimprinted PVDF–MWCNT Composites with Silver Coating as a Label-Free Plasmonic Platform for Ultrasensitive Detection

**DOI:** 10.1021/acsanm.4c06987

**Published:** 2025-04-08

**Authors:** Aeshah F. Alotaibi, Waseem Ahmad Wani, Ghadeer Almohammadi, Brian J. Rodriguez, James H. Rice

**Affiliations:** † School of Physics, University College Dublin, Belfield, Dublin 4 D04 C1P1, Ireland; ‡ Department of Physics, College of Science and Humanities, Shaqra University, Shaqra 11961, Kingdom of Saudi Arabia; § Conway Institute of Biomolecular and Biomedical Research, University College Dublin, Belfield, Dublin 4 D04 C1P1, Ireland; ∥ School of Chemistry, University College Dublin, Belfield, Dublin 4 D04 C1P1, Ireland; ⊥ Department of Chemistry, College of Science, University of Hafr Al-Batin, Hafr Al-Batin 39524, Saudi Arabia

**Keywords:** surface-enhanced Raman scattering (SERS), nanoimprinting, polyvinylidene fluoride (PVDF), multiwalled carbon nanotubes (MWCNTs), plasmonic nanostructures, localized surface plasmon resonances (LSPR), atomic force microscopy (AFM)

## Abstract

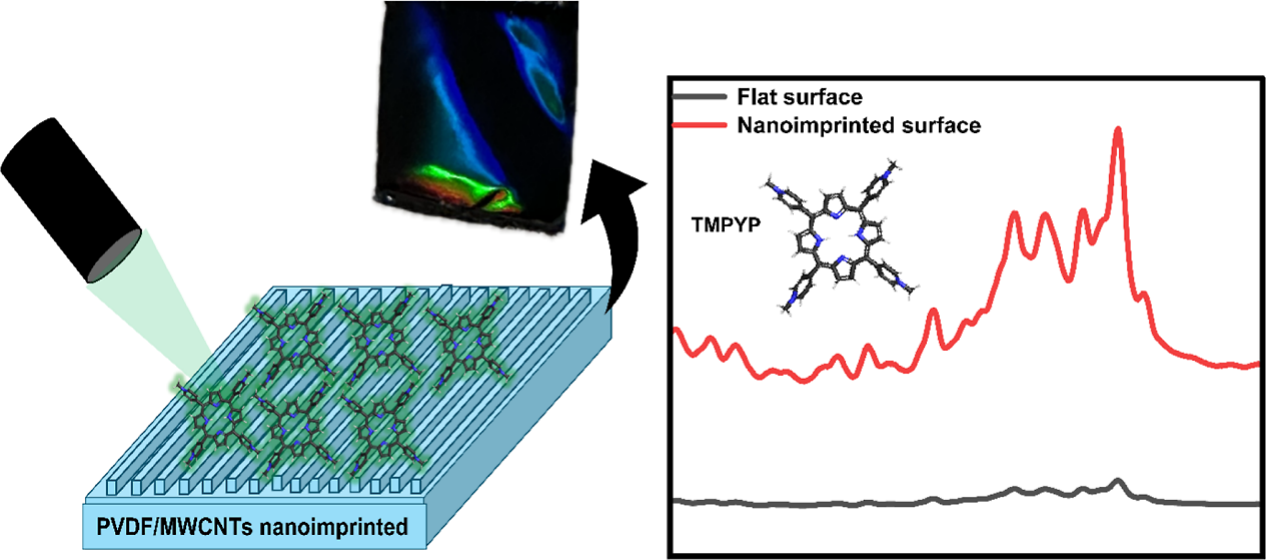

This study investigates the development of an organic hybrid composite based on polyvinylidene fluoride and multiwalled carbon nanotubes. We examine the use of nanoimprinting to form submicron arrays with roughened surface topography on the surface of the organic composite. This nanoscale surface roughening effect arises from the nanotubes at the surface, which is absent in imprinted pure polyvinylidene fluoride. We demonstrate that depositing a thin silver layer onto the nanoimprinted organic composite containing multiwalled carbon nanotubes (MWCNTs) produces an approximately 10-fold enhancement in surface-enhanced Raman scattering (SERS) signal intensity compared to the nanoimprinted pure PVDF film and also yields a significantly higher SERS response than that observed on a flat (nonimprinted) MWCNT/PVDF composite. Furthermore, subjecting the nanoimprinted MWCNT/PVDF composite to superband gap irradiation results in an additional 2-fold increase in SERS signal strength. This research highlights the potential of a polymer doped with carbon nanotubes to form plasmon active arrays for sensitive and efficient chemical detection.

## 1 Introduction

All-organic composites are of significant research interest in regard to forming stable, robust, and lightweight materials. Polyvinylidene fluoride (PVDF) is a highly versatile thermoplastic fluoropolymer known for its excellent chemical resistance, high purity, and unique physical properties.^1−5^ Through the formation of composites, a range of physical properties for PVDF can be enhanced. The incorporation of carbon nanotubes (CNTs) into polyvinylidene fluoride (PVDF) matrices is known to enhance mechanical, electrical, and thermal properties.^1−4^ PVDF-based nanocomposites are gaining traction for applications in energy harvesting and sensing.^5^ For example, the strategic arrangement of CNTs into hierarchical nanoarrays can enhance the stability and conductivity of composite materials, which is crucial for demanding applications.^6^ Research shows that the percolation threshold for achieving conductivity can be as low as 0.1 wt %, allowing for effective electrical performance even at minimal carbon nanotube content.^2^ The thermal properties are further improved by the presence of carbon nanotubes, making them suitable for applications involving high-temperatures such as following exposure to laser irradiation.^7^ Additionally, adding carbon nanotubes to PVDF significantly increases the ultimate tensile strength and elastic modulus of the composite material.^8^

Soft nanoimprinting forms nanometer-scale patterns using a mold-based methodology.^9−12^ This approach is particularly advantageous for producing high-resolution patterns cost-effectively and with high throughput. This approach relies on the mechanical deformation of an imprint resist—typically a polymer. By controlling nanoscale geometries, custom spectral responses can be engineered.^9^ Through imprinting submicrometric periodic lattices onto a polymer surface, photonic resonances can be formed in these structures, occurring due to the interaction of light with the periodic arrangement of the imprinted nanostructured surface features. This interaction leads to constructive and destructive interference of light waves, which enhances specific wavelengths. The ability to control these resonances allows for tunable optical properties. Through the application of a thin (ex 10 nm) plasmon active metal coating, plasmonic crystals can be created. These crystals enhance light–matter interactions, making them suitable for applications such as surface-enhanced Raman spectroscopy (SERS). Through utilization of electromagnetic hotspots engineered with regular spacing by the imprinting process, sensitive and reproducible SERS detection can be enabled.^9−14^

The surface roughness of a plasmon active substrate significantly influences the sensitivity of SERS. Rough surfaces create localized electromagnetic fields, which allow for effective coupling of the incident light, leading to increased SERS signal intensity from molecules adsorbed on these surfaces.^15−18^ An ideal SERS substrate should have a specific roughness that balances enhancement with reproducibility. Achieving optimal surface roughness in similar organic material systems is crucial for surface light interactions,^19^ but excessive roughness can lead to nonuniform signal enhancement and inconsistent spectral data, while insufficient roughness limits enhancement.^15^

Studies of the role of surface roughness on the SERS performance of imprinted plasmonic architectures reported that roughed plasmonic crystal substrates outperform their smooth (unroughened) counterparts regarding the Raman enhancement factor (EF).^15,16^ These studies reported that in the roughed substrates, a larger population of localized surface plasmon resonances (LSPR) triggered at the sharp edges and gaps of the rough metal surface. These studies report increases in EF of up to 10^3^ for roughened surfaces compared to the absence of surface roughness.^15,16^ Here, we investigate the development of a nanoimprinted silver-coated organic hybrid composite based on polyvinylidene fluoride and multiwalled carbon nanotubes. We also assess the impact of the introduction of carbon nanotubes in the formation of a roughened surface topography on the nanoarray structures and the impact of this roughened surface on SERS signal strength. This research highlights the potential of a polymer doped with carbon nanotubes to form plasmon active arrays for sensitive and efficient chemical detection.

## 2 Experimental Section

### 2.1 Preparation of PVDF–MWCNT Nanocomposite Films

**Figure 1:**
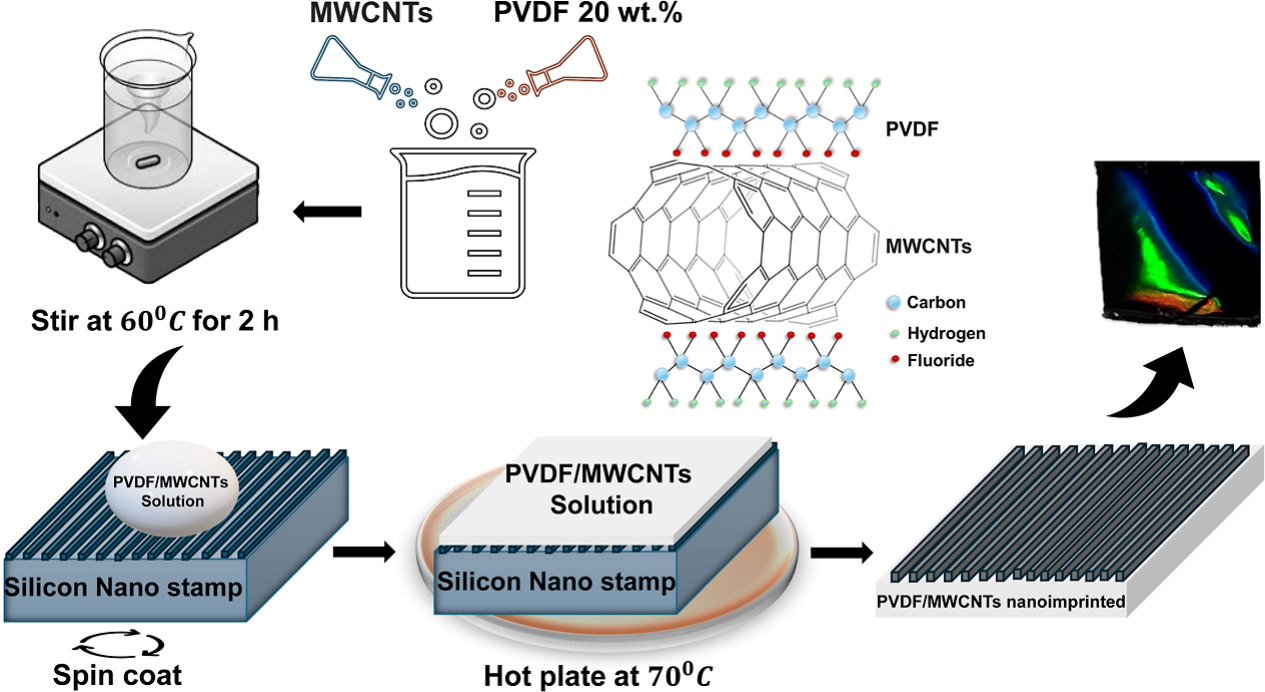
Schematic representation of the synthesis process for PVDF–MWCNT nanocomposites and the methodology used for imprinting. A schematic drawing of a nanotube and PVDF monomers is also shown, indicating that the π-electron-rich surface of MWCNTs enables effective interactions with polar regions of PVDF.

The synthesis of piezoelectric thin films for atomic force microscopy (AFM) analysis involved a novel approach utilizing PVDF-based nanocomposites. The process began with the preparation of a 20% PVDF solution in DMF, which underwent thorough agitation in a heated bath at 60 °C for 60 min to ensure homogeneity. This solution was then mixed with varying concentrations of multiwalled carbon nanotubes (MWCNTs) at 0.5, 2, 3, and 5 wt %. The mixture was stirred at 60 °C for an additional 2 h. The resulting solution was spin-coated onto a linear silicon nanostamp at 1 V for 30 s to form an even layer. After coating, the substrate was annealed at 70 °C on a hot plate, and the PVDF–MWCNT film was carefully peeled off, retaining the nanoscale features of the stamp, as shown in Figure 1.

### 2.2 Probe Molecule Solutions Preparation

To prepare the probe molecule solutions, T (meso-tetra (*N*-methyl-4-pyridyl) porphine tetrachloride) (TMPyP), crystal violet (CV), and methylene blue (MB) powder were dissolved in purified water to achieve an initial molarity of 10^–1^. This solution was further diluted using deionized water to reach a final concentration of 10^–5^ M. The resulting solution was then carefully applied to the surface of PVDF and PVDF/MWCNT nanoimprinted samples using a drop-casting technique.

### 2.3 Analytical Techniques

#### 2.3.1 Surface-Enhanced Raman Spectroscopy

SERS measurements were conducted using a sophisticated Raman system. The setup comprised a spectrograph (SP2300i, Princeton Instruments), a CCD camera (iDus 401, Andor), and a green laser source (532 nm wavelength, 5 mW power, MGL-III-532 model). The system also incorporated a beam splitter and long-pass filter (RazorEdge, Semrock). For imprinted samples, the presented data represents an average of approximately 30 individual measurements.

#### 2.3.2 Atomic Force Microscopy

AFM imaging was performed using an MFP-3D instrument from Asylum Research, operating in amplitude modulation mode. The imaging process utilized monolithic silicon probes (Tap300Al-G) featuring an aluminum reflective coating. These probes were characterized by a force constant range of 20–75 N/m (40 N/m nominal), resonance frequencies between 200 and 400 kHz (300 kHz nominal), and lengths varying from 115 to 135 μm (125 μm nominal). For measuring the contact potential difference (CPD) of PVDF and PVDF/MWCNT composites, the **PPP-EFM** AFM probes were utilized. This combination of probes and techniques enabled a comprehensive analysis of the samples’ topographical and electrical properties.

#### 2.3.3 UV–Visible Reflection Spectroscopy

Specular reflectance measurements were obtained using a LAMBDA 750 UV/vis/NIR spectrophotometer from PerkinElmer. An aluminum mirror served as the reference standard for calculating relative reflectance changes. The spectral data acquisition covered a wavelength range of 250–1000 nm, with measurements taken at 1 nm intervals.

#### 2.3.4 Fourier Transform Infrared Spectroscopy

Fourier transform infrared spectroscopy (FTIR) analysis was conducted using a NICOLET iS50 FT-IR spectrometer, providing comprehensive spectral information for the samples under investigation.

#### 2.3.5 Dynamic Mechanical Analysis

The dynamic mechanical analysis (DMA) for the PVDF/MWCNT composites was performed using the NETZSCH DMA 242-E instrument, a highly precise device for analyzing the mechanical and viscoelastic properties of materials. This instrument provided accurate measurements of the storage modulus, damping behavior, and other essential parameters, enabling a detailed evaluation of the composites’ performance.

## 3 Results and Discussion

**Figure 2:**
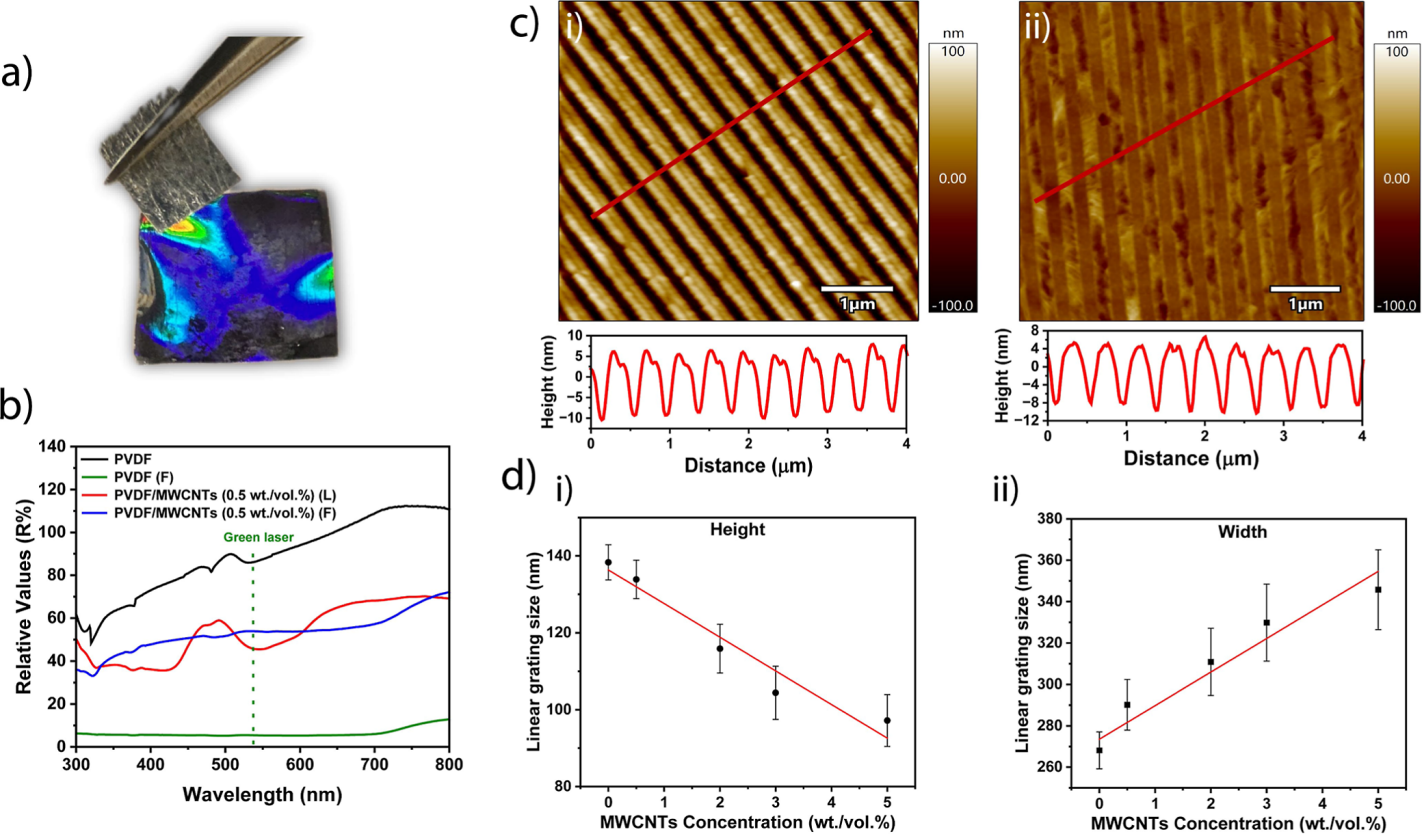
(a) Photograph of a PVDF–MWCNT (0.5 wt vol %) thin film showing light diffraction created by the film. (b) Reflection spectra for an imprinted (labeled L) PVDF–MWCNT (0.5 wt vol %) that is not imprinted (labeled F). Reflection spectra for the imprinted film showing bands at c.a. 500 and 675 nm and dips at c.a. 400 and 550 nm. (c) AFM topography imaging of (i) pure PVDF thin film and (ii) PVDF–MWCNT (0.5 wt vol %) thin film. (d) Plots of substrate grating feature size vs MWCNT concentration, showing that as the nanotube concentration increases, the width and height of the grating topography increase and reduce, respectively. Note that the same nanostamp is being used to prepare all the samples. A linear fit was made to the data (red line), showing a linear correlation with feature size and MWCNT concentration.

In this study, nanocomposites incorporating polyvinylidene fluoride (PVDF) were synthesized using a solvent-based nanoimprinting technique. In brief, a PVDF solution was mixed with multiwalled carbon nanotubes (MWCNTs) at 5% weight/volume. The resulting solution was spin-coated onto a linear silicon nanostamp, after which the PVDF–MWCNT film was carefully peeled off, retaining the nanoscale features of the stamp, as shown in Figure 1. Following the imprinting process, the PVDF–MWCNT film was adorned with a 10 nm layer of silver (Ag), a plasmon-active metal. The imprinted PVDF–MWCNT film, now transformed into an optical diffraction grating, exhibited striking structural coloration due to the nanostructures gracing its surface (Figure 2a).

The reflection spectrum of the imprinted PVDF–MWCNT 0.5% weight/volume thin film following silver coating was then recorded (Figure 2b). The reflectance spectrum shows dips around 425 and 550 nm. These dips indicate plasmonic Bragg modes arising from the submicron grating period, leading to constructive and destructive interference of different diffraction orders.^2,10−12^ This results in the coloration of the substrate as shown in Figure 2a. The impact of the nanotubes on the surface topography was analyzed using atomic force microscopy (AFM) (Figure 2c(ii)) for the imprinted PVDF–MWCNT 0.5% weight/volume thin film sample. The AFM image of silver-coated and imprinted pure PVDF film shows a relatively smooth surface morphology (Figure 2c(i)). An AFM line scan over the surface of the PVDF–MWCNT 0.5% weight/volume composite shows a markedly rougher surface compared to pure PVDF (Figure 2c). The surface topography is regular for the imprinted pure PVDF sample, with a small variation in the imprinted surface features, while in contrast, the addition of 0.5% weight/volume MWCNTs to form the PVDF–MWCNT composite increases the surface heterogeneity (Figure 2c), introducing additional nanoscale textures on the imprinted surface features.

A series of PVDF–MWCNT thin films were then made with increasing concentrations of MWCNTs (from 0.5 to 5% weight/volume). AFM topography images were recorded for each of these samples (Supporting Information Figures S1–S4). Examining the AFM topography images scan shows that the introduction of increasing concentrations of MWCNTs (from 0.5 to 5% weight/volume) changes the feature size of the resulting grating (which was created using the same nanostamp). At a MWCNT concentration of 0.5% weight/volume, the grating possesses a width and height of 270 and 137 nm, respectively (Figure 2d(i and ii)). As more MWCNT is introduced into PVDF, these dimensions change linearly with nanotube concentration (Figure 2d). At a MWCNT concentration of 5% weight/volume, the grating possesses a width and height of 340 and 100 nm, respectively. This change in the topography of the resulting polymer–nanotube composites may be due to the presence of the nanotubes causing a swelling of the polymer, increasing the grating features width while reducing its height. These changes in feature size was accommodated by changes in the reflection spectra (Supporting Information Figure S5a). The spectral dip in the reflection spectra observed for a MWCNT concentration of 0.5% weight/volume at ca. 550 nm shifts sequentially with increasing nanotube concentration to ca. 500 nm for a MWCNT concentration of 5% weight/volume, in agreement with AFM topography measurements.

**Figure 3:**
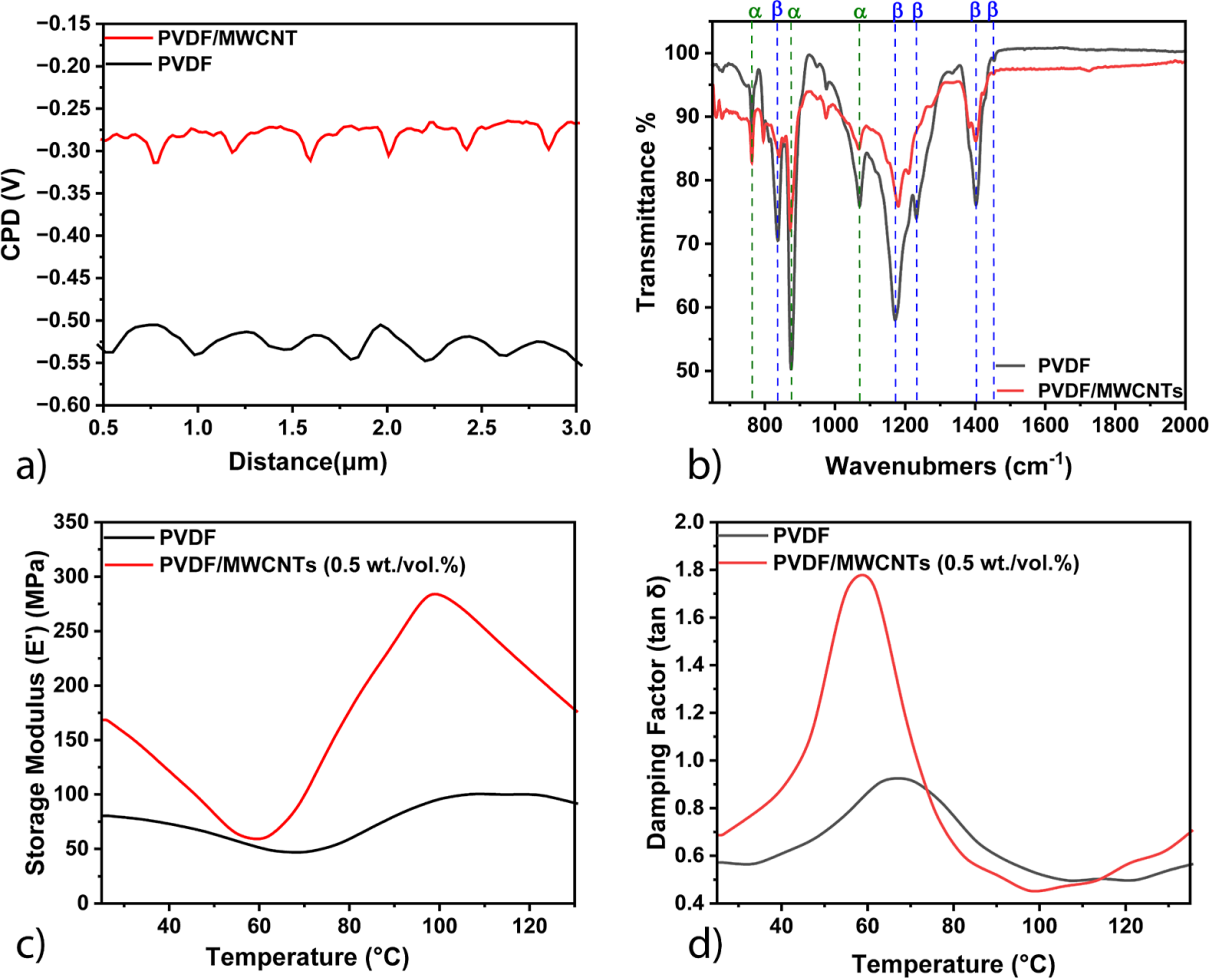
(a) Contact potential difference (CPD) of PVDF and PVDF–MWCNT (0.5% weight/volume). The data show that the composite possesses a higher CPD value in comparison to pure PVDF. (b) FTIR spectra of PVDF and PVDF–MWCNT (0.5% weight/volume). Indicated on the spectra are the band positions associated with beta phase (blue lines) and alpha phase (green lines). (c) Storage modulus of PVDF and PVDF–MWCNT (0.5% weight/volume) as a function of temperature. (d) Damping factor of PVDF and PVDF–MWCNT (0.5% weight/volume).

Surface potential measurements (Figure 3a) show that the PVDF/MWCNT (0.5% weight/volume) composite exhibits a higher surface potential compared to pure PVDF. Pure PVDF has a contact potential difference (CPD) of −0.50 V, while PVDF mixed with 0.5% weight/volume MWCNTs has a CPD of −0.28 V. A less negative CPD indicates a higher work function. The work function can be influenced by the interaction between the polymer and the nanotubes. A higher work function in such a composite could suggest that the interaction between the nanotubes and the polymer is strong, potentially leading to better charge distribution across the composite.^2,20^ The interaction between the MWCNTs and PVDF is primarily driven by physical entanglement and noncovalent interactions such as van der Waals forces and π–π stacking.^1,2,21^ The π-electron-rich surface of MWCNTs enables effective interactions with polar regions of PVDF, culminating in a more homogeneous blend.^1,21^ The FTIR spectra (Figure 3b) show characteristic α-phase peaks at approximately 761 cm^–1^and β-phase peaks near 837 cm^–1^. The FTIR spectrum for the PVDF–MWCNT (0.5% weight/volume) composite shows that the β-phase is present at an amount lower than that for pure PVDF. Specifically, the β-phase content for pure PVDF is 66.61%, while for the PVDF–MWCNT (0.5% weight/volume) composite, it is 37.88%. In contrast, the α-phase content for pure PVDF is 33.42%, and for the PVDF–MWCNT (0.5% weight/volume) composite, it is 62.13%.

The analysis of the storage modulus (Figure 2c) for PVDF and the PVDF/MWCNT (0.5% weight/volume) composite reveals significant insights into their mechanical behavior across a range of temperatures as the storage modulus is a measure of the elastic stiffness of a material, indicating how well the material resists deformation under stress. Pure PVDF exhibits a relatively stable storage modulus (ca. 75 MPa at room temperature), which changes slightly with increasing temperature. In contrast, the PVDF/MWCNT (0.5% weight/volume) composite exhibits a modulus of ca. 175 MPa at room temperature. Increasing the MWCNT concentration up to 5% weight/volume increases the storage modulus to ca. 750 MPa (Supporting Information Figure S5b). This shows that at room temperature, the storage modulus increases by ca. a factor of 10 when a large concentration of nanotubes (e.g., 5% weight/volume) is introduced. The composite with 0.5% weight/volume MWCNTs shows an initial increase in modulus, peaking around 100 °C before declining. This suggests enhanced stiffness due to the reinforcing effect of MWCNTs, likely attributed to improved load transfer within the matrix.^2,20^ Higher concentrations of MWCNTs (2, 3, and 5% weight/volume) consistently exhibit increased stiffness compared to pure PVDF, particularly at room temperatures, indicating that MWCNTs contribute significantly to the mechanical reinforcement of the composite. Figure 3d illustrates the damping factor (tan δ) as a function of temperature for both pure PVDF and the PVDF–MWCNT (0.5% weight/volume). The damping factor in composites is influenced by the properties of the matrix, the reinforcement material, and the quality of the interaction between these components. The graph shows that the composite exhibits a higher damping factor than pure PVDF, indicating improved energy dissipation capabilities. This enhancement is likely due to increased interfacial interactions between PVDF and MWCNTs, which facilitate better energy absorption and dissipation.^2,22^ The peak in the damping factor occurs around 80 °C, suggesting optimal energy dissipation at this temperature.^2^

**Figure 4:**
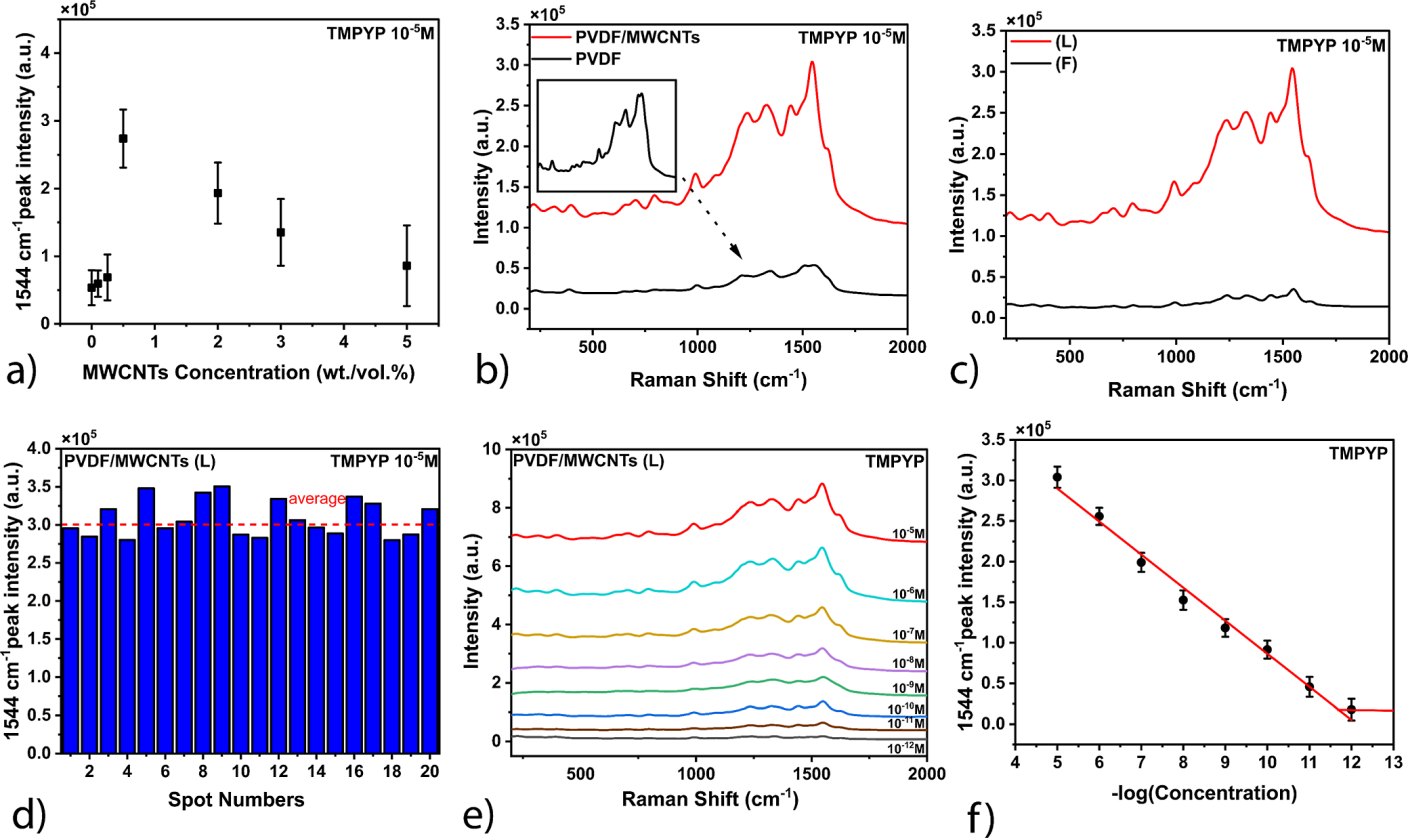
TMPYP Raman spectra. (a) Signal intensity for PVDF/MWCNTs with varying MWCNT concentrations. (b) Comparison of TMPYP spectra for PVDF and PVDF/MWCNTs. (c) TMPYP spectra for PVDF/MWCNTs in linear (L) and flat (F) forms. (d) Histogram showing TMPYP signal stability across 20 spots. (e) TMPYP spectra at different concentrations. (f) Linear plot of TMPYP signal intensity vs −log(concentration).

Silver-coated PVDF–MWCNT (0.1 to 5 wt/vol %) thin films were evaluated as SERS substrates, with the results presented in Supporting Information Figure S6. The Raman spectra indicate that as the MWCNT concentration increases, the characteristic D (∼1325 cm^–1^) and G (∼1575 cm^–1^) bands of the nanotubes become more pronounced.^1^ However, the SERS spectra reveal that 0.5 wt/vol % MWCNT loading provides the highest enhancement for the probe molecule TMPYP, as shown in Figure 4a. At lower concentrations (0.1 and 0.25 wt/vol %), signal enhancement is still observed, but it is weaker due to the insufficient density of MWCNTs, which limits the formation of effective plasmonic hotspots after silver coating.^1,23^

At 0.5 wt/vol %, the balance between hotspot density and background suppression is optimized, leading to the most prominent enhancement of TMPYP peaks at ∼1240 cm^–1^ (C-pyrrole bending), ∼1450 cm^–1^, and ∼1544 cm^–1^ (C–C stretching).^1,23,24^ The SERS enhancement from silver-coated PVDF–MWCNT (0.1 to 5% weight/volume) thin films is likely due to optimal electromagnetic field localization, consistent with studies showing that controlled filler concentrations can improve SERS performance by optimizing hotspot distribution.^25^ However, at higher MWCNT concentrations (2–5 wt/vol %), the increased presence of carbon nanotubes contributes to a stronger intrinsic Raman background, reducing the visibility of the probe molecule’s peaks. This phenomenon has been widely reported, as excessive carbon-based fillers can suppress SERS effects by masking analyte signals due to dominant nanotube-related Raman scattering.^24,25^ These findings reinforce the importance of fine-tuning MWCNT loading in composite materials to optimize SERS sensitivity for molecular detection applications. Figure 4b compares the SERS performance between PVDF and PVDF/MWCNTs, where the addition of MWCNTs significantly enhances the Raman signal approximately 6-fold (peak to peak ratio). This improvement is attributed to MWCNTs’ ability to increase the surface roughness and introduce plasmonic hotspots, enhancing the electromagnetic field, which is critical for SERS signal amplification.^24^ Furthermore, Figure 4c demonstrates that the linear imprinted geometry shows about a 6-fold increase in SERS performance compared to the flat surface, which is consistent with the enhanced alignment of plasmonic hotspots in anisotropic structures.^26^

Figure 4d demonstrates the stability of the SERS signals by plotting the intensity at 1544 cm^–1^ across 20 different spots on the PVDF/MWCNT substrate. The minimal variation in signal intensity across the different spots confirms the reliability and reproducibility of the substrate for SERS applications. Figure 4e,f highlights the substrate’s sensitivity, showing that PVDF/MWCNTs can detect TMPYP at picomolar concentrations, with a linear relationship between the signal intensity at 1544 cm^–1^ and −log(concentration), confirming its potential for trace detection applications. This sensitivity aligns with previous research that underscores the effectiveness of MWCNT-based composites in detecting low-concentration analytes due to their large surface area and high electrical conductivity.^2^

In Figure S7a,b Supporting Information, the SERS performance for crystal violet (CV) and methylene blue (MB) is compared using PVDF/MWCNT substrates in linear and flat geometries. The linear geometry consistently shows superior enhancement across multiple peaks, especially those located around 1170 cm^–1^ (C–H bending), 1375 cm^–1^ (C–N stretching), and 1620 cm^–1^ (aromatic ring stretching) for CV,^27^ and 450 cm^–1^ (skeletal vibrations of MB), 1395 cm^–1^, and 1625 cm^–1^ for MB.^1,28^ The improved signal for the linear structure is likely due to the increased surface area and enhanced charge transfer properties, leading to stronger Raman scattering from the molecules. This is further supported by the more effective localization of the electromagnetic field in the linear geometry, which creates more plasmonic hotspots, essential for maximizing SERS activity.

**Figure 5:**
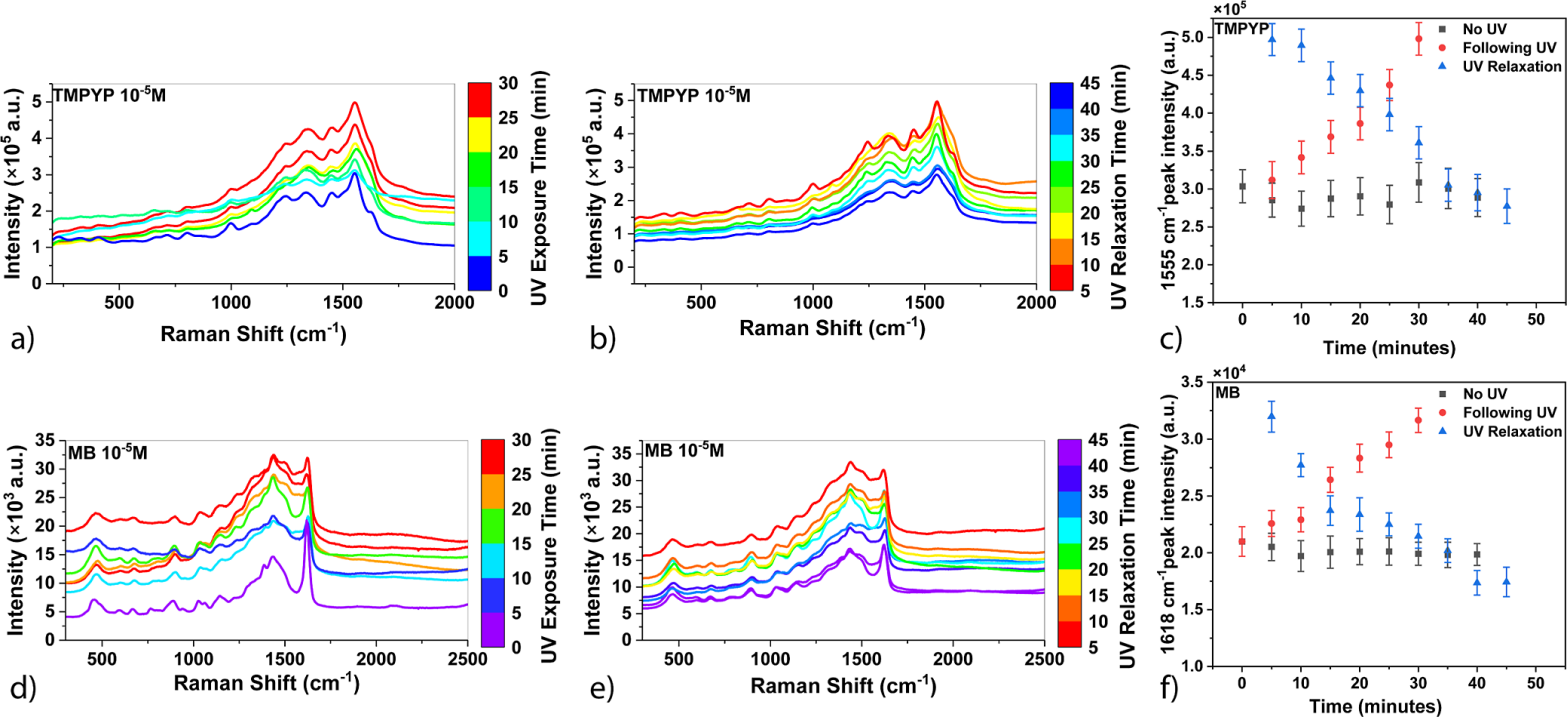
(a) TMPYP spectra with UV exposure. (b) TMPYP spectra during UV relaxation. (c) TMPYP peak intensity comparison over time: UV exposure, relaxation, and no UV. (d) MB spectra with UV exposure. (e) MB spectra during UV relaxation. (f) MB peak intensity comparison over time: UV exposure, relaxation, and no UV.

Figure 5 demonstrates the effects of UV exposure and relaxation on the SERS performance of TMPYP (Figure 5a–c) and methylene blue (MB) (Figure 5d–f) using PVDF/MWCNT substrates. In Figure 5a, TMPYP spectra show increased intensity with UV exposure, indicating enhanced electromagnetic interactions. Conversely, Figure 5b reveals that during relaxation, TMPYP intensity decreases over time, suggesting a temporary enhancement effect. Figure 5c compares the peak intensity of TMPYP under different conditions: UV exposure, relaxation, and no UV treatment. The results indicate that UV exposure provides the highest enhancement, while relaxation leads to a decline in intensity. Figure 5d–f confirms these trends for MB, demonstrating that UV-induced enhancement is temporary and diminishes over time. This aligns with studies showing that UV irradiation can enhance SERS by optimizing plasmonic hotspot distribution.^1,29^

**Figure 6:**
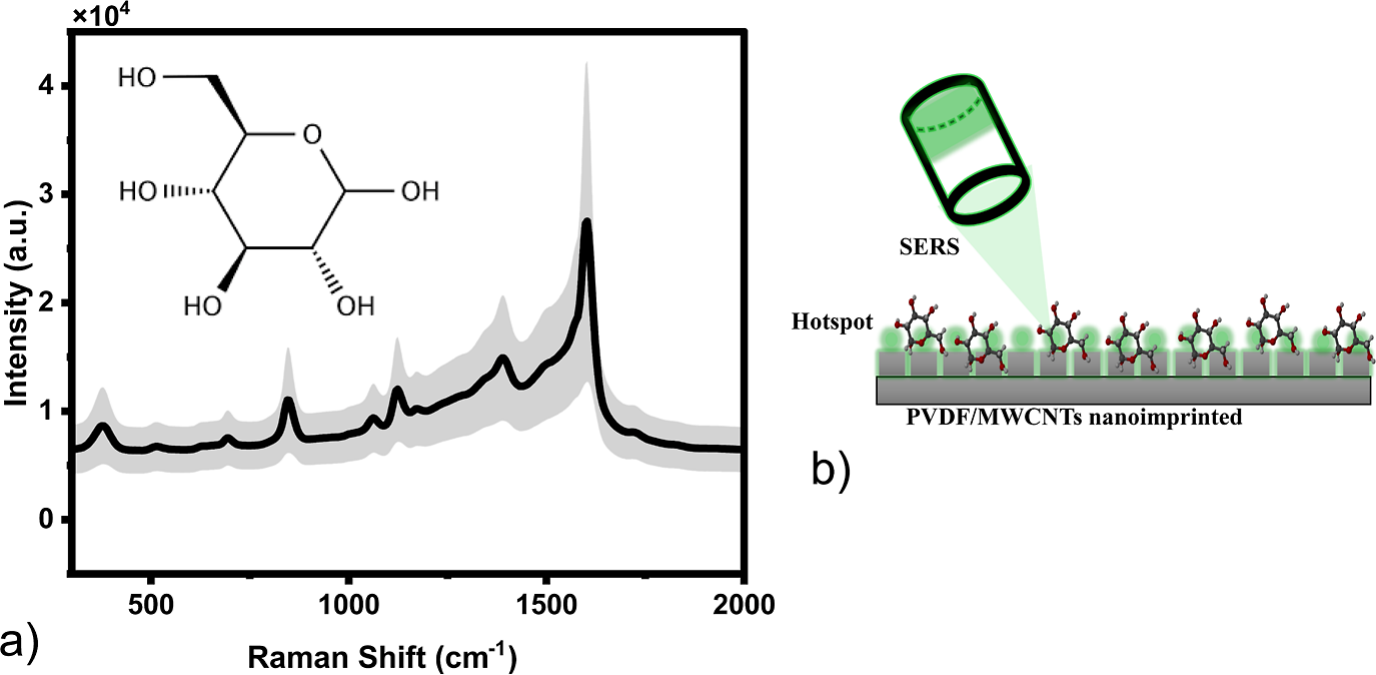
(a) Raman spectrum of glucose (10^–5^ M) on the nanoimprinted PVDF/MWCNTs substrate. (b) Schematic of the nanoimprinted PVDF/MWCNTs substrate, highlighting hotspots for SERS enhancement.

The Raman spectrum obtained from the PVDF/MWCNTs nanoimprinted substrate (Figure 6a) demonstrates the effective enhancement of glucose signals at a low concentration of 10^–5^ M. Key Raman peaks observed near 500 cm^–1^, 900 cm^–1^, and 1500 cm^–1^ correspond to the ring-breathing, C–O–C stretching, and C–H bending modes characteristic of glucose molecules.^29^ The high intensity observed in the spectrum, reaching up to 4 × 10^4^ a.u., indicates significant enhancement attributed to surface-enhanced Raman spectroscopy (SERS).^30^ This enhancement arises from localized electromagnetic hotspots on the nanoimprinted substrate, which amplify the Raman scattering of glucose molecules adsorbed on the surface.^28^ The ability of the PVDF/MWCNTs substrate to detect such low concentrations of glucose aligns with previous studies, which demonstrate that SERS can achieve high sensitivity even at concentrations as low as 10^–5^ M.^31−33^

The schematic diagram (Figure 6b) of the PVDF/MWCNTs substrate highlights the nanoimprinted structure that facilitates reproducible and efficient SERS activity. The integration of MWCNTs into the PVDF matrix enhances the substrate’s conductivity and surface area, while the nanoimprinting process ensures the uniform formation of electromagnetic hotspots.^1,12^ These hotspots are crucial for amplifying Raman signals, especially in the detection of biomolecules like glucose at low concentrations. Studies on similar PVDF/MWCNT-based SERS substrates have shown that nanoimprinting improves signal consistency and detection sensitivity by creating well-defined nanostructures that maximize the local electromagnetic fields. This combination of PVDF’s mechanical stability with the enhanced conductivity of MWCNTs makes the substrate a promising tool for sensitive biomolecular detection.

## 4 Conclusion

The development of nanoimprinted PVDF-MWCNT composites has yielded promising results for SERS applications. The synergistic effects of PVDF’s mechanical properties and MWCNTs’ electrical conductivity, combined with nanoimprinting techniques, have resulted in self-energized SERS substrates with enhanced sensitivity and efficiency. The composites demonstrated improved surface roughness, higher surface potential, and optimized light absorption characteristics, all contributing to superior SERS performance. The ability to detect various analytes, including biomolecules like glucose, at low concentrations showcases the potential of these materials for applications in environmental monitoring, biomedical diagnostics, and food safety testing. The linear nanoimprinted structures consistently outperformed flat geometries, highlighting the importance of controlled nanostructuring in SERS substrate design. Future research should focus on further optimizing MWCNT concentrations and exploring additional nanoimprinting patterns to maximize SERS enhancement and expand the range of detectable analytes. The self-energizing nature of these composites opens up new possibilities for portable and field-deployable SERS devices, potentially revolutionizing on-site chemical and biological sensing technologies.

## Supporting Information Available

Additional AFM images of the PVDF/MWCNT composites at 0.5, 2, 3, and 5 wt/vol %; reflection spectra and storage modulus data for PVDF and PVDF/MWCNT composites at various concentrations; TMPYP Raman spectra PVDF/MWCNTs with varying MWCNT concentrations; crystal violet (CV) and methylene blue (MB) spectral comparisons for linear versus flat PVDF/MWCNT structures; and UV–vis absorbance spectra of PVDF/MWCNT composites (with varying concentrations) together with that of pure MWCNTs (PDF)
