# Supplementary material for: Nanoimprinted PVDF–MWCNT Composites with Silver Coating as a Label-Free Plasmonic Platform for Ultrasensitive Detection
Source: ACS Appl Nano Mater. 2025 Apr 8;8(15):7440–8. doi: 10.1021/acsanm.4c06987 (PMC12012746; doi:10.1021/acsanm.4c06987)
Supplement: Supplementary file 1 — an4c06987_si_001.pdf [file an4c06987_si_001.pdf]

Supporting Information

Nanoimprinted PVDF–MWCNT Composites with Silver Coating as a Label-Free Plasmonic Platform for Ultrasensitive Detection.

Aeshah F. Alotaibi[1,2*]; Waseem Ahmad Wani[3]; Ghadeer.Almohammadi[4,5]; Brian J. Rodriguez[1,3]; James H. Rice[1*]

[1] School of Physics, University College Dublin, Belfield, Dublin 4, Ireland
[2] Department of Physics, College of Science and Humanities, Shaqra University, Shaqra, Kingdom of Saudi Arabia
[3] Conway Institute of Biomolecular and Biomedical Research, University College Dublin, Belfield, Dublin 4, Ireland
[4] School of Chemistry, University College Dublin, Belfield, Dublin 4, Ireland
[5] Department of Chemistry, College of Science, University of Hafr Al-Batin, Hafr Al-Batin, Saudi Arabia.

Email: afal-otaibi@su.edu.sa; james.rice@ucd.ie

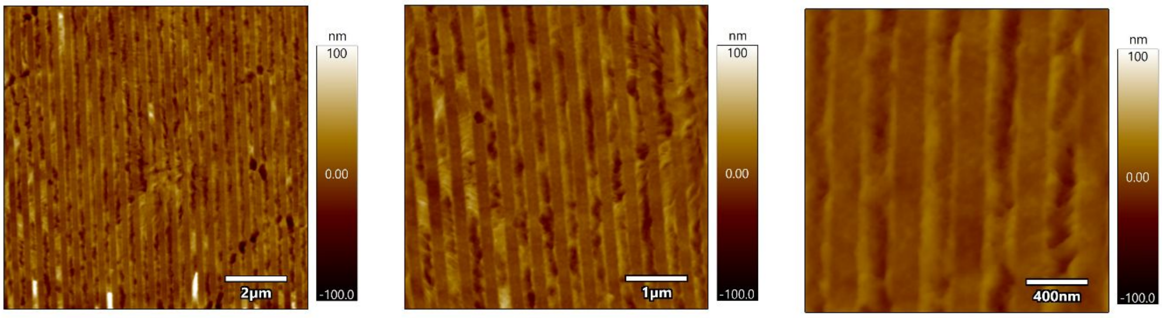

Figure S 1: Atomic Force Microscopy (AFM) images of PVDF/MWCNT composite at 0.5 wt./vol.% concentration.

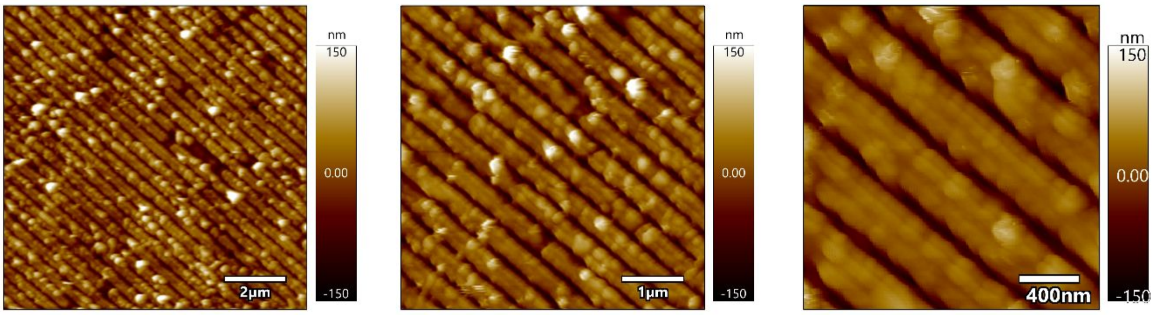

Figure S 2: Atomic Force Microscopy (AFM) images of PVDF/MWCNT composite at 2 wt./vol.% concentration.

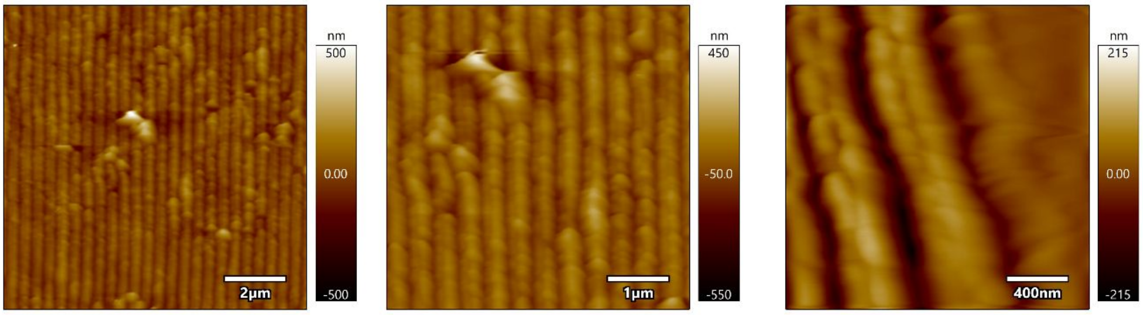

Figure S 3: Atomic Force Microscopy (AFM) images of PVDF/MWCNT composite at 3 wt./vol.% concentration.

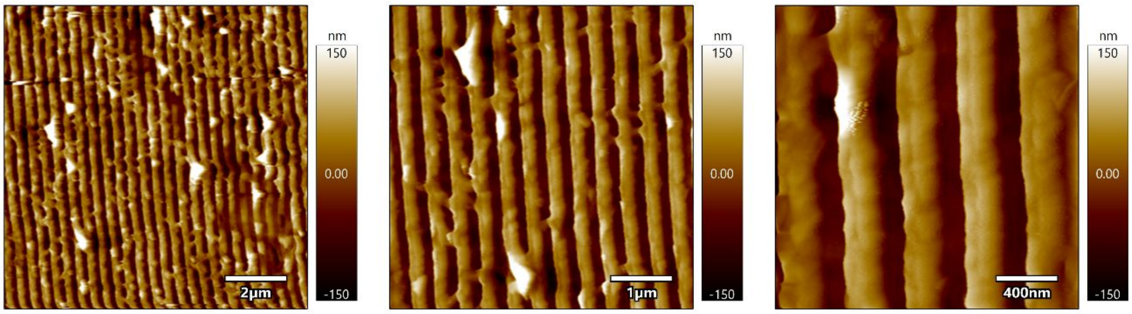

Figure S 4: : Atomic Force Microscopy (AFM) images of PVDF/MWCNT composite at 5 wt./vol.% concentration.

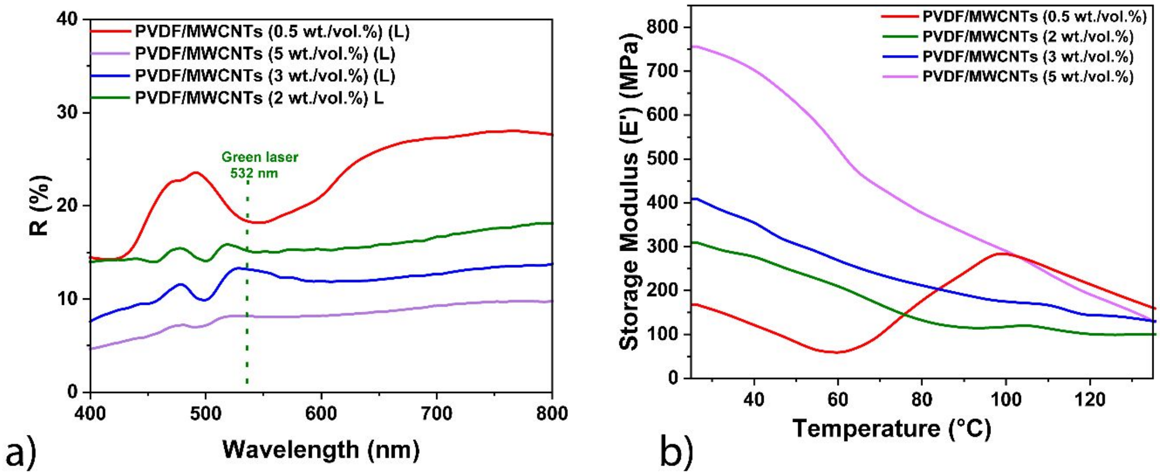

Figure S 5: a) reflection spectra of PVDF and PVDF/MWCNT at different concertation. b) storge modulus of PVDF/MWCNT at different concertation.

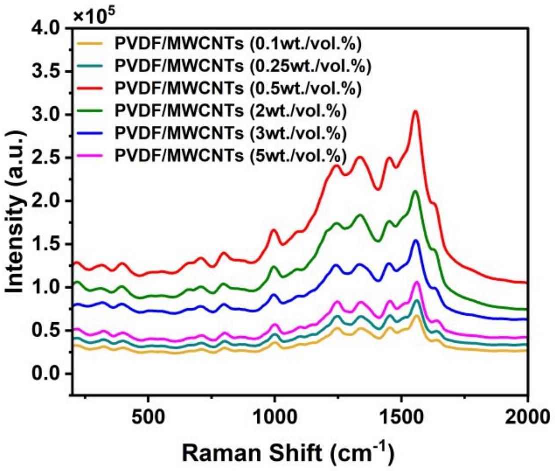

Figure S 6: TMPYP Raman spectra, PVDF/MWCNTs with varying MWCNT concentrations.

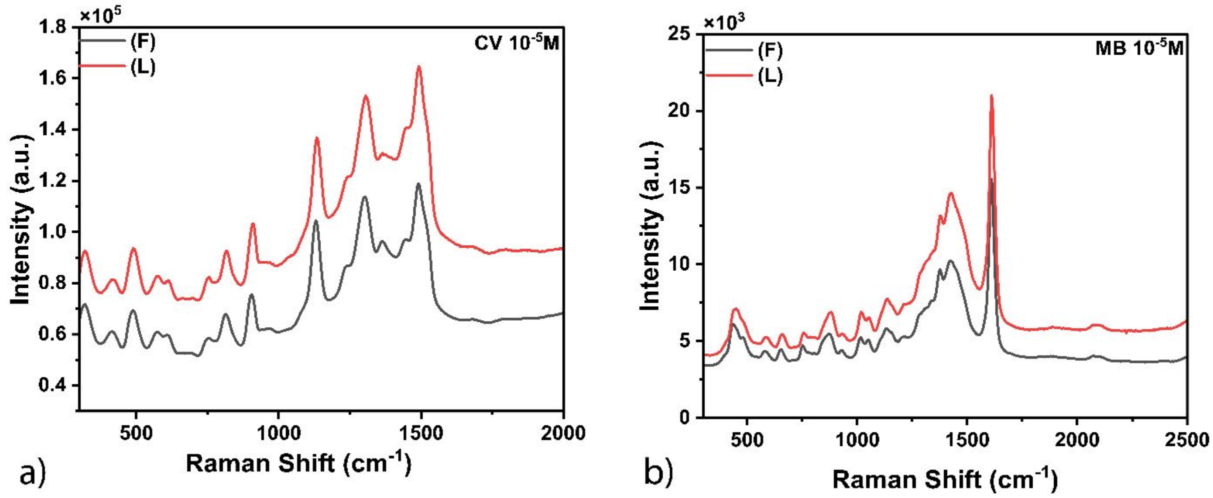

Figure S 7 :a) CV spectra for linear and flat PVDF/MWCNTs. b) MB spectra for linear and flat PVDF/MWCNTs.

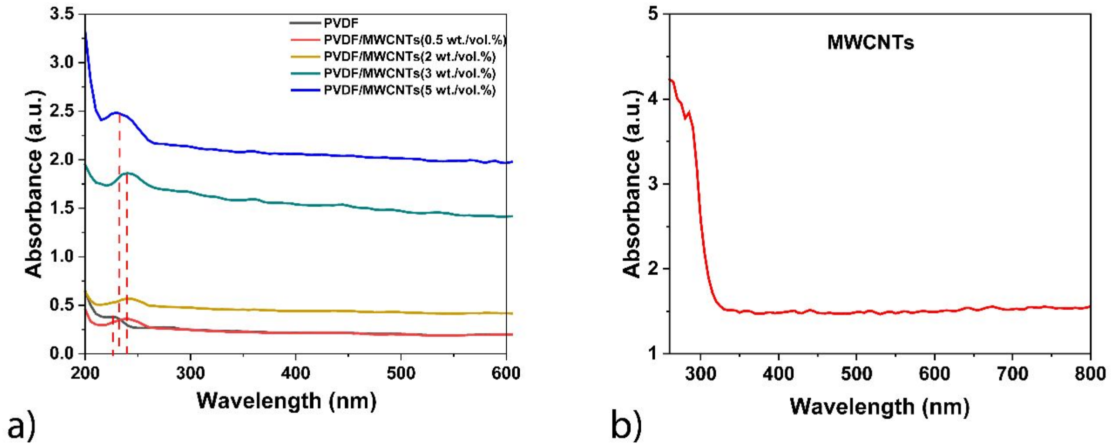

Figure S 8: a) UV-Vis Absorbance Spectra of PVDF and PVDF/MWCNT composites with varying concentrations. b) UV-Vis Absorbance Spectrum of pure MWCNTs
